# Case Report: E-cadherin and Vimentin expression in advanced-stage pancreatic ductal adenocarcinoma tissue and circulating tumor cells: preliminary results from a pilot study

**DOI:** 10.3389/fonc.2026.1770280

**Published:** 2026-05-29

**Authors:** Maria Cristina Rapanotti, Maria Giovanna Scioli, Alessandro Moscardelli, Edoardo Troncone, Elisa Cugini, Tara Mayte Suarez Viguria, Lorena Vergilii, Martina Puzzuoli, Silvia Anzillotti, Sonia Terriaca, Tonia Cenci, Eleonora Muzzi, Elena De Cristofaro, Luca Savino, Luca Toti, Anastasia De Luca, Amedeo Ferlosio, Giovanna Del Vecchio Blanco, Augusto Orlandi

**Affiliations:** 1 Anatomic Pathology, Department of Integrated Care Processes, Policlinico Tor Vergata, Rome, Italy; 2 Anatomic Pathology, Department of Biomedicine and Prevention, University of Rome Tor Vergata, Rome, Italy; 3 Gastroenterology Unit, Department of Systems Medicine, University of Rome Tor Vergata, Rome, Italy; 4 Hepatobiliary Surgery and Transplant Unit, Department of Surgical Sciences, University of Rome "Tor Vergata", Rome, Italy; 5 Laboratory Medicine, Department of Integrated Care Processes, Policlinico Tor Vergata, Rome, Italy; 6 Department of Biology, University of Rome Tor Vergata, Rome, Italy

**Keywords:** pancreatic ductal adenocarcinoma, endoscopic ultrasound fine-needle biopsy, epithelial to mesenchymal transition, circulating tumor cells, E-cadherin expression, Vimentin expression

## Abstract

**Background:**

Pancreatic ductal adenocarcinoma (PDAC) has a dismal prognosis due to late diagnosis and early metastasis. The lack of robust biomarkers necessitates new strategies to stratify high-risk patients. Endoscopic ultrasound-guided fine-needle biopsy (EUS-FNB) is the gold standard for tissue acquisition. Concurrently, circulating tumor cells (CTCs) offer a non-invasive tool for prognostic assessment, specifically regarding the epithelial-to-mesenchymal transition (EMT). The loss of E-cadherin and the expression of Vimentin are key hallmarks of this transition, facilitating a metastatic phenotype and CTC survival.

**Aim and objectives:**

This retrospective, preliminary pilot study of 11 patients with advanced PDAC at onset aimed to investigate the expression of EMT markers in EUS-FNB tissue biopsies. Concurrently, we characterized EMT profiles across isolated PDAC CTCs comprising —CD45^-^EPCAM^+^CD74^+^, CD45^-^EPCAM^-^CD74^+^, and CD45^-^EPCAM^+^CD74^-^ subpopulations—from the same patients. The inclusion of CD74-positive cells was prioritized due to the role of CD74 not only as an invasive marker but also as a functional driver of the epithelial-mesenchymal transition in cancer.

**Methods:**

EUS-FNB was performed for histological diagnosis and IHC analysis of E-cadherin and Vimentin in 11 PDAC samples (grade G1–G3). Concurrently, CTC subpopulations were isolated via immunomagnetic CD45 depletion and EpCAM/CD74 enrichment. E-cadherin and Vimentin transcript levels in CTCs were then quantified using real-time PCR.

**Results:**

Among the 11 PDAC EUS-FNB biopsies, E-cadherin expression was absent in three cases, all of which were G3 tumors. The majority of samples (72.7%) exhibited low-to-moderate E-cadherin positivity. Regarding Vimentin, biopsies were generally negative, with the exception of small tumor cell clusters (<5%) identified in G3 samples. In contrast, E-cadherin transcripts were not detected in the corresponding CTC subpopulations. Vimentin expression was observed in the majority of CTC samples (81.8%), with nine cases showing moderate-to-high levels and the remaining two cases exhibiting faint expression.

**Conclusions:**

Our assay demonstrated a potentially favorable performance, highlighting distinct EMT expression between the primary tumor and CTCs. This baseline comparison of EMT markers in matched PDAC samples and CTCs underscores the significance of mesenchymal-invasive profiles, despite the limited cohort size.

## Introduction

Pancreatic ductal adenocarcinoma (PDAC) shows a survival rate of less than 5% and a median survival of less than 1 year. The only potentially curative treatment for PDAC is surgical resection; however, more than 80% of PDAC patients are unresectable (1). Diagnosis in advanced stages with local invasion or distant metastasis is due to the lack of clinically informative early diagnostic symptoms.

Endoscopic ultrasound (EUS) with fine-needle biopsy (FNB) is currently the preferred minimally invasive procedure to obtain tissue for histopathological diagnosis in suspected pancreatic lesions. The relevance of EUS-FNB lies in the possibility of obtaining core tissue useful not only for histopathological diagnosis of malignancy but also for immunehistochemical staining and molecular evaluations (2–4).

Considering the high frequency of the metastatic disease at the onset of PDAC, the identification of biomarkers that allow for the selection of patients with more aggressive disease is necessary to guide the choice of the most appropriate treatment for each patient.

Many studies have shown that many factors, including epithelial-mesenchymal transition (EMT), immunehistochemical staining, and other specialized tests compared to cell clusters obtained from fine-needle aspiration (FNA) (4, 5), the tumor microenvironment (6, 7), and circulating tumor cells (CTCs) (8, 9), play vital roles in pancreatic cancer metastasis. Specifically, regarding CTCs, upon detachment from primary tumors, these cells degrade the extracellular matrix (ECM) and secrete proteolytic enzymes, facilitating migration and intravasation into the bloodstream. These cells possess information about the genomic characteristics of their tumor source *in situ*, and their detection and characterization hold great promise for personalized cancer management. These epithelium-derived cancer cells are thought to undergo morphological changes associated with EMT (8, 10). At metastatic sites, neoplastic cells must also infiltrate the endothelium in order to colonize new tissues and be able to induce neoangiogenesis to ensure sufficient blood supply to the newly formed tumor. A reverse behavioral change, mesenchymal-to-epithelium transition (MET), takes place at metastatic sites. Several genes related to EMT are usually detected in tumor metastasis and CTCs. Recently, Vimentin and E-cadherin have been shown to be prognostic markers in some malignant tumors, where notable changes have been reported. The loss of E-cadherin-mediated adhesion plays an important role in the transition of epithelial tumors from a benign to an invasive state. Vimentin, a mesenchymal type III intermediate filament, is also functionally associated with pro-metastatic functions, including tumor cell migration and CTC survival (9, 11).

Loss of intercellular adhesion and increased cell motility promote tumor cell invasion. In the present study, E-cadherin and Vimentin are investigated as inducers of EMT, which is thought to play a fundamental role during the early steps of invasion and metastasis of carcinomas. The purpose of this study was to investigate whether a gain in Vimentin and loss of E-cadherin expression in advanced pancreatic cancer are involved in the process of metastasis via EMT.

In PDAC, Rhim et al., 2012 (4) found that pancreatic cancer cells undergo EMT early during cancer development. Microenvironmental changes in pancreatic cancer are the primary causes of EMT, the key event leading to invasion and metastasis.

Our group previously investigated “stem-mesenchymal” circulating cells in melanoma using an approach that combined an in-house immunomagnetic enrichment procedure with molecular profiling (12–17). In the present study, we evaluated a dual-targeting positive selection for CTCs by simultaneously targeting the epithelial marker EpCAM (CD326) and the CD74 epitope. Given its association with perineural invasion (18, 19) and its role as a potential EMT driver in PDAC and other malignancies (20–22), the inclusion of anti-CD74 was intended to facilitate the capture of CTC subpopulations with a mesenchymal phenotype, which may lack epithelial markers following transition. Our findings suggest that combining these epithelial, mesenchymal, and invasion biomarkers could potentially address the challenge of capturing heterogeneous PDAC-CTCs that may harbor clinical information.

### Purpose

In this retrospective, preliminary pilot study, we analyzed the immunohistochemical expression of EMT by evaluating E-cadherin and Vimentin markers in PDAC EUS-FNB biopsies. Furthermore, we characterized the “mesenchymal-invasive” profile of isolated PDAC CTCs—comprising CD45^-^EPCAM^+^CD74^+^, CD45^-^EPCAM^-^CD74^+^, and CD45^-^EPCAM^+^CD74^-^—from the same patient by performing a molecular expression assay of these two key EMT markers. The aim of the present pilot study was to investigate the effect of microenvironmental changes on the epithelial−mesenchymal transition (EMT) in PDAC (23).

Correlations among the protein expression of E-cadherin and Vimentin in tissues were compared to their corresponding molecular expression in the reported PDAC CTCs. The inclusion of CD74-positive cells was prioritized due to the dual role of CD74 as an invasive marker and as an EMT functional driver.

## Materials and methods

In total, 11 treatment-naïve patients were considered eligible if they had a histological EUS-FNB-confirmed PDAC diagnosis. We retrieved formalin-fixed paraffin-embedded (FFPE) EUS-FNB PDAC tumor biopsies obtained at diagnosis and collected peripheral blood samples (15mL) for PDAC CTC recovery by immunomagnetic enrichment. Patients’ demographic, clinical, and histological characteristics are shown in **Table 1**. Information and consent forms, previously approved by the local Institutional Review Board (CET 06/10/2023 prot. PTV 0022164/2023), were provided at diagnosis, together with authorization to use tissue biopsies and collect blood samples for research purposes.

**Table 1:** Patients’ demographic, biological, histological and clinical characteristics.

Patient data	N	%
**Total patients**	**11**	
**Sex** Female Male	5 6	45.45 54.54
**Tumor location** Head/Uncinate/neck Body/Tail	8 3	72.72 27.27
AJCC* (American Joint of Cancer Committee) Stage **III** **IV**	2 9	18.1 81.8
**Metastatic Spread** LN metastases Distant metastases	4 7	36.36 63.63
**Somatic hallmark-mutation *status* on FFPE-EUS-FNB** *KRAS* codon 12 *BRCA1 and BRCA2*	11 0	100 0
**Desmoplasia** Absent Present	4 7	36.36 63.63

^*^ The AJCC staging was evaluated at onset.

Moreover, we defined the grade of each biopsy classifying it according to the standard scale from G1 to G3, where G1 indicates low grade (well differentiated and less aggressive), G2 indicated moderate grade, and G3 indicates high grade (poorly differentiated and more aggressive).

Immunohistochemistry (IHC) was used to detect and quantify E-cadherin and Vimentin protein expression in the 11 formalin-fixed paraffin-embedded tissue sections for EMT characterization. An EUS-FNB PDAC sample was defined as a tumor when ≥50% tumor cells were detected. Immunohistochemical staining was performed using a standard avidin-biotin-peroxidase complex procedure. Briefly, five-µm-thick sections were cut and subjected to antigen retrieval using citrate buffer (pH 6.0). The sections were then incubated with primary antibodies anti-Vimentin (Abcam Inc., Cambridge, UK, ab8978, 1:200) and anti-E-cadherin (ab1416, 1:100) for 1 h at room temperature, followed by incubation with specific secondary antibodies. Immunohistochemical reaction was visualized with 3,3’diaminobenzidine (DAB) as the substrate (24). External positive and negative controls were previously used to validate the IHC protocol. Two investigators simultaneously assessed all tissue sections in a blinded manner. The immunohistochemical signal positivity was scored following an established protocol (11): 0 for background staining, 1 for faint staining, 2 for moderate staining, and 3 for strong staining. In addition, the percentage of positive cells was graded according to the following scale: 0 for < 5%, 1 for 5–25%, 2 for 26–50%, 3 for 50–75%, and 4 for 75–100%. These two parameters were multiplied to produce a weighted score for each specimen (intensity × percentage of positive cells); a sample was considered “positive” only if the weighted score was > 3, with expression levels further categorized into low (3–5), moderate (6–8), and high (>9) expression. Samples with a weighted score was < 3 were considered negative.

CTC enrichment was performed on 15 mL peripheral blood samples collected from the same cohort of 11 patients. After removing platelets and erythrocytes via density-gradient centrifugation, CTC subpopulations were isolated using a sequential immunomagnetic approach. This consisted of an initial negative selection for CD45^+^ leukocytes, followed by an in-house dual-positive enrichment targeting EpCAM (CD236) (BD Pharmingen™, San Diego, CA, USA) and CD74 (Biorbyt™ Cambridge, UK), according to the manufacturer’s instructions (EasySep Magnetic Nanoparticles, StemCell; BD Pharmingen™, San Diego, CA, USA). The stoichiometry and experimental conditions for antibody-cell conjugation are reported previously (12–17). Dual-targeting positive selection with these two antibodies enabled the enrichment of a heterogeneous population of PDAC CTCs, comprising distinct subpopulations, such as CD45^-^EPCAM^+^CD74^+^, CD45^-^EPCAM^-^CD74^+^, and CD45^-^EPCAM^+^CD74^-^.

To standardize the molecular analysis, a semi-quantitative scoring system was first established through spike-in experiments using titrated pancreatic and mesenchymal cell lines (12–17). This standardized scale was then applied to evaluate the 11 PDAC CTC samples. Specifically, total RNA was extracted from the patient-derived CTC subpopulations, and E-cadherin and Vimentin transcript levels were quantified by qRT-PCR (normalized to ACTB)(25). Based on the thresholds defined by the spike-in model, qRT-PCR values (arbitrary units, a.u.) were categorized into four groups: negative (<2.5 a.u.), low (2.6–110 a.u.), moderate (111–600 a.u.), and high (>600 a.u.).

### Statistical analysis

For statistical evaluations, given the limited sample size, we stratified the 11 EUS-FNB PDAC samples into two grading categories: well/moderately differentiated (low-grade, G1-G2) and poorly differentiated (high-grade, G3). These categories were compared with respect to E-cadherin and Vimentin protein expression (**Table 2a**). Concurrently, we evaluated the molecular expression of E-cadherin and Vimentin transcripts in the corresponding CTC subpopulations (**Table 2b**). Data were analyzed using X-square test and Fisher’s exact tests. Given the exploratory nature of this study, p-values are reported to indicate the strength of the observed associations, with *p* < 0.05 used as a conventional reference point for descriptive purposes.

**Table 2:** Association between protein expression of E-cadherin/Vimentin in PDAC FFPE-EUS-FNB samples (a) and molecular expression of *E-cadherin*/*Vimentin* in PDAC CTC subpopulations (b).

UPN COD	sex	Age at onset	Primary PDAC site	AJCC	Grading	a) Tissue expression in PDAC FFPE-EUS-FNB		b) mRNA expression in PDAC CTC-subpopulations: *CD45^-^EPCAM^+^CD74^+^* *CD45^-^EPCAM^-^CD74^+^* *CD45^-^EPCAM^+^CD74^-^*	
						E-cadherin	Vimentin	*E-cadherin*	*Vimentin*
UPN1-AG	F	68	HEAD	III	G3	loss/absence	negative	negative	moderate
UPN2-VE	F	55	HEAD	IV	G2	moderate	negative	negative	high
UPN3-PM *	M	68	TAIL	IV	G3	low	negative	negative	low
UPN4-RG *	M	79	HEAD	IV	G3	low	negative	negative	low
UPN5-CA	F	78	BODY	III	G3	low	negative	negative	moderate
UPN6-DGLM	M	64	HEAD	IV	G1/G2	moderate	negative	negative	high
UPN7-AM	M	60	HEAD	IV	G1/G2	moderate	negative	negative	high
UPN8-PA	F	62	HEAD	IV	G3	low	negative	negative	high
UPN9-TS	F	39	HEAD	IV	G1/G2	moderate	negative	negative	moderate
UPN10-AG	M	70	HEAD	IV	G3	loss/absence	negative	negative	high
UPN11-BV	M	56	HEAD	IV	G3	loss/absence	negative	negative	high

*PDAC CTC subpopulations sharing low molecular Vimentin expression.

In addition, patient-specific clinicopathological features are reported.

## Results

### EMT marker expression in PDAC tissue biopsies

**Figure 1:**
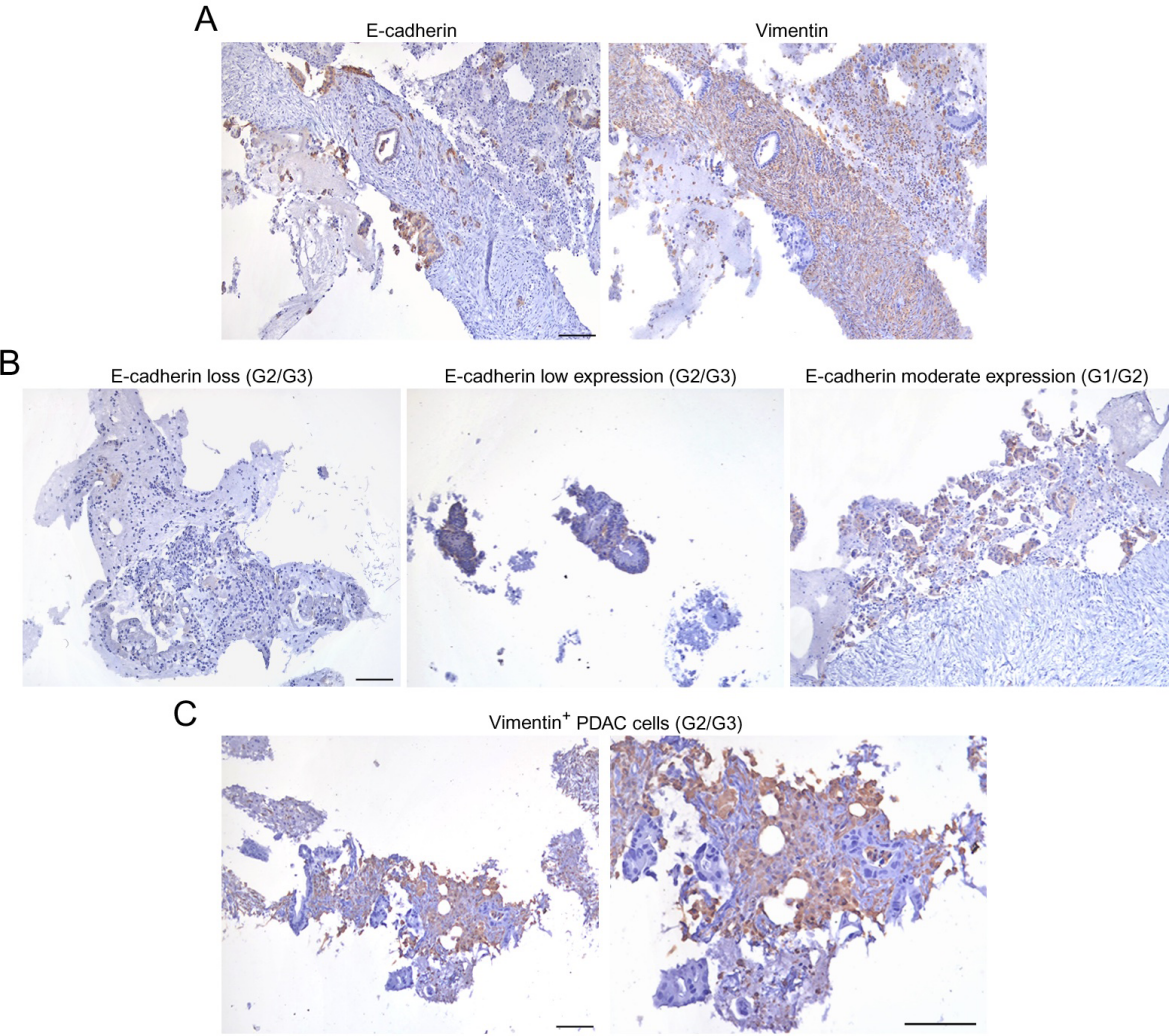
Expression of EMT markers E-cadherin and Vimentin in PDAC EUS-FNB tissue biopsies. **(A)** Immunohistochemical (IHC) staining of a PDAC sample showing E-cadherin expression in tumor cells, while Vimentin is localized only in the surrounding stroma (internal positive control). **(B)** Representative image of two poorly differentiated G2/G3 PDAC biopsies displaying a loss/absence of E-cadherin expression compared with higher E-cadherin expression in G1/G2 PDAC biopsy. (C) G2/G3 PDAC sample showing small, scattered tumor cell clusters (<5%) expressing Vimentin; these cases were classified as negative according to the weighted score threshold (<3).

Among the 11 PDAC samples analyzed, only three cases revealed no expression/loss of the epithelial marker E-cadherin in tumor cells. The majority of samples demonstrated low-to-moderate positivity of tumor cells that were negative for Vimentin. The latter was highly expressed in the surrounding tumor stroma, serving as a positive internal control (**Figure 1A**).

Interestingly, E-cadherin expression was associated with tumor differentiation grade. Loss or low E-cadherin expression was mainly observed in G3 samples, whereas only G1/G2 samples showed a moderate expression (**Table 2a**, **Figure 1B**). Only small tumor cell clusters in G3 samples expressed Vimentin (**Figure 1C**); however, these samples were considered negative because the score was below 3 (positivity cutoff). Immunohistochemical comparison of E-cadherin expression between the two grading categories (low and high grade) demonstrated a statistically significant association (p<0.0009).

### Molecular gene expression in a heterogeneous population of PDAC CTCs

Gene expression analysis in PDAC-CTCs subpopulations showed molecular expression at the limit of detection for the *E-cadherin* gene in 4 out of 11 PDAC CTC-subpopulations (36.34%, expression score 0.007-1.7 a.u.); therefore, they were all considered negative (0 for < 2.5 a.u).

*Vimentin* molecular expression was detected in all CTC samples (100%, expression score 31.48–7311 a.u). In 9 out of 11 patients, moderate-to-high expression levels were observed, whereas two CTC patients (UPN2 and UPN3, marked with an asterisk in **Table 2**) showed low molecular *Vimentin* expression (expression score >2.6–110<a.u).

In our small cohort, there was a negative association between *E-cadherin* and *Vimentin* expression within PDAC CTC subpopulations (p<0.0001; **Table 2b**).

## Conclusions

EMT, characterized by a gain of mesenchymal cell markers and a loss of epithelial markers (26, 27), is a process whereby cells acquire molecular alterations that facilitate cell motility and invasion (26, 28, 29).

During EMT, cancer cells lose the ability to recognize specific targets and usually develop autocrine loops for growth factors, which play essential roles in providing self-sustaining growth signals to cancer cells. Through EMT, tumor cells acquire features of mesenchymal cells (30, 31). It has been reported that high Vimentin and reduced E-cadherin expression are important predictors across several types of cancers, such as gastric, colorectal, and pancreatic cancers. Particularly, the mRNA expression of epithelial markers, such as *E-cadherin*, decreases, whereas that of mesenchymal markers, such as *Vimentin*, increases during EMT (32–35). The loss of *E-cadherin* has been reported to be associated with poor clinical outcome (36–38).

On the other hand, Vimentin expression in epithelial cancer cells has been shown to be associated with metastasis and poor survival in several cancers (39), including PDAC (9, 40–43).

Moreover, Vimentin and E-cadherin have also been shown to be prognostic markers. In particular, it has been reported that acquisition of contextual Vimentin expression and loss of E-cadherin result in tumor metastasis, invasion, radioresistance, and generation of cancer cells with stem cell-like characteristics in neuroendocrine pancreatic cancer (11). The aim of this retrospective, preliminary pilot study was to observe potential variations in EMT hallmarks in advanced PDAC by analyzing E-cadherin and Vimentin expression in EUS-FNB tissues and matched circulating tumor cells.

Given the absence of established clinical biomarkers for this aggressive pathology, our study explored whether CTCs could provide additional insights into the EMT process.

Our group previously focused on the development of an “in-house” protocol that “mimics” as closely as possible the condition of circulating minimal residual disease in solid tumor, allowing the detection of circulating tumor cells (15–17). While our previous findings in melanoma showed a strong clinical association, the results presented here for PDAC suggest a potential role for CTCs as a non-invasive tool, although their prognostic value remains to be fully established in this context.

Molecular expression of *Vimentin* and *E-cadherin* might also serve as a biomarker panel that can aid in the prognostication of patients.

We decided to enrich and isolate an epithelial/mesenchymal phenotype by targeting the epithelial marker EpCAM, the *pan* “epithelial-cell-adhesion-molecule” (CD326), and the CD74 epitope, a marker associated with the “perineural invasion” and an EMT driver (18, 19–22, 44). 

This preliminary pilot study documented that in 3 out of 11 EUS-FNB PDAC samples (27.7%), E-cadherin expression was absent, resulting in *loss-negative*, and 4 out of 11 samples (36.36%) showed only low expression; All of these tissue biopsies were staged as G3, which means poorly differentiated PDAC. The remaining four biopsies showed moderate E-cadherin and were graded G1/G2 (well to moderately differentiated). All EUS-FNB PDAC samples included in this preliminary pilot study did not express Vimentin. This finding is noteworthy, especially because our small cohort consisted exclusively of locally-advanced PDAC (AJCC stage III-IV); nevertheless, we documented the absence of Vimentin expression in PDAC tissue, if not revealed for small scattered tumor cell clusters in G2/G3 PDAC samples. This finding contrasts with other cancers, such as pancreatic neuroendocrine tumors, where *high* Vimentin expression and *loss* of E-cadherin (absence) expression in patient biopsies have been associated with significantly elevated risks of lymph node metastasis, distant metastasis, perineural invasion, and advanced American Joint Committee on Cancer (AJCC) stage compared with the other expression groups (11).

The analysis of the matched counterparts heterogeneous PDAC CTC subpopulations (CD45^-^EPCAM^+^CD74^+^, CD45^-^EPCAM^-^CD74^+^, and CD45^-^EPCAM^+^CD74^-^) revealed an absence of *E-cadherin* gene expression, whereas moderate-to-high *Vimentin* levels were detected in the majority of samples. These preliminary observations indicate differences in EMT marker profiles between the primary tissue and the circulating compartment. However, these findings are exploratory and necessitate further validation through larger prospective cohorts and longitudinal follow-up to better characterize PDAC CTC dynamics and their clinical significance.

These findings concur with evidence that *loss* (absence) or *downregulation* of *E-cadherin* expression results in the subsequent progression of cells toward a malignant phenotype. Conversely, *Vimentin* plays a fundamental role in tumor invasion and metastasis. CTCs that undergo EMT, such as the hybrid “mesenchymal-invasive” PDAC CTC subpopulations, exhibit significant changes in the cell phenotype through downregulation of *E-cadherin* and acquisition of key mesenchymal markers, such as *Vimentin*.

Despite the limited cohort size, this pilot study provides preliminary observations regarding the expression of E-cadherin and Vimentin in advanced-stage PDAC (45–47). Pancreatic cancer is a rapidly progressive disease, and early diagnosis remains challenging. Early pancreatic cancer diagnosis is a rare event since symptoms usually appear late, when tumors have fully progressed. EMT is an important molecular mechanism underlying early invasion and metastasis in PDAC (48–50).

The absence of Vimentin in PDAC tissue does not imply an indolent nature; rather, it reflects differences in EMT marker profiles between tissue and circulating compartments, with the latter exhibiting heightened aggressiveness during cell dissemination. These preliminary findings, while limited by the pilot nature of the study, highlight the unique dynamic of PDAC and underscore the clinical relevance of CTCs in capturing the metastatic potential of the disease.

Our findings suggest that PDAC may exhibit a distinct spatial EMT dynamic, in which mesenchymal markers are more prominent in the circulating compartment than in the primary tumor tissue. Although the consistent loss of E-cadherin in G3 cases was observed, its definitive prognostic value in this setting requires further confirmation.

To refine our understanding of EMT hallmarks and transcription factors, future prospective studies should prioritize expanding the cohort size and molecular profiling of PDAC, including pancreatic neuroendocrine tumor (PNET) tissues. Refinement of CTC isolation using markers such as Cell-Surface Vimentin (CSV) and investigating of diverse E-cadherin expression patterns will be essential. While EMT-associated CTCs—particularly hybrid subpopulations—may offer diagnostic and prognostic insights, these findings require validation in larger prospective studies before they can be considered reliable tools for monitoring disease status or predicting treatment response.

EUS-FNB represents an effective methodology for obtaining high-quality PDAC tissue samples, enabling reliable downstream analysis.

## Data Availability

The raw data supporting the conclusions of this article will be made available by the authors, without undue reservation.
